# Validation of Different Methods of Preparation of *Adhatoda vasica* Leaf Juice by Quantification of Total Alkaloids and Vasicine

**DOI:** 10.4103/0250-474X.40329

**Published:** 2008

**Authors:** S. Soni, Sheetal Anandjiwala, G. Patel, M. Rajani

**Affiliations:** Pharmacognosy and Phytochemistry Department B. V. Patel Pharmaceutical Education Research Development Centre (PERD), Thaltej, Ahemdabad - 380 054, India

**Keywords:** *Vasaka* juice, total alkaloids, vasicine

## Abstract

Leaf of *Adhatoda vasica* (*Vasaka*) is an important drug of Ayurveda, prescribed as an expectorant. Quinazoline alkaloids present in the leaves are established as active principles. In Ayurveda, its leaf juice (*Vasa swarasa*) is incorporated in many formulations. Classical method for extracting the juice (*swarasa*) from the leaf is an elaborate process, which involves subjecting a bolus of crushed fresh leaf to heat followed by squeezing out the juice. Commercially, to prepare the juice of *Vasaka*, manufacturers have been adopting different methods other than the traditional method. In an effort to evaluate these modified processes phytochemically to identify the process which gives juice of the quality that is obtained by traditional method, in terms of its alkaloid content, we prepared the leaf juice by traditional Ayurvedic method, its modification by steaming of leaf to simulate the traditional method and other methods adopted by some manufacturers. These juice samples were evaluated for the total alkaloid content by spectrophotometric method and vasicine content by thin layer chromatography densitometric method using high performance thin layer chromatography. The high performance thin layer chromatography method was validated for precision, repeatability and accuracy. The total alkaloid content varied from 0.3 mg/ml to 5.93 mg/ml and that of vasicine content varied from 0.2 mg/ml to 5.64 mg/ml in the juice samples prepared by different methods. The present study revealed that steaming of fresh leaves under 15 lb pressure yielded same quantity of juice as the traditional bolus method (25 ml/100 g leaf) and its total alkaloid content and vasicine content (4.05±0.12 and 3.46±0.06 mg/ml, respectively) were very high when compared to the other methods, though the traditional method was found to give the best quality juice with highest amount of total alkaloids (5.93±0.55 mg/ml) and vasicine (5.64±0.10 mg/ml) content.

*Adhatoda vasica* Nees. leaf (*Vasaka*), known as *Vasa* in Ayurveda, is an important drug prescribed for malarial fever, fever caused by *pitta* and *kapha*, chronic fever, intrinsic hemorrhage, cough and asthma, leprosy, skin diseases and piles^1^. It is reported to be an expectorant^2^, abortifacient^3^, antimicrobial^4^,^5^, antitussive^6^ and anticancer^7^. Important chemical constituents of leaf include pyrroloquinazoline alkaloids, vasicine (fig. 1), vasicol, adhatonine, vasicinone, vasicinol, vasicinolone^8^. Vasicine was reported to have bronchodilatory, respiratory stimulant and uterine stimulant effect^9^. Vasicinone was shown to have bronchodilatory, weak cardiac stimulant and antianaphylactic action^10^. In Ayurvedic preparations, *Vasaka* leaf juice (*Vasa swarasa*) is incorporated in more than 20 formulations including *Vasarishta*, *Mahatiktaka ghrita*, *Triphala ghrita*, *Vasavaleha*, *Vasakasava*, *Mahatriphalaghrita*, *Panchatiktaghritaguggulu* and *Panchatikta ghrita*^11^. Classical method for extracting *Vasaka* juice is an elaborate process which involves subjecting a bolus of crushed fresh leaf to heat^12^. This method is not applicable in large scale extraction of juice for commercial purpose. Hence, in the commercial manufacture of the formulations containing *Vasaka* juice (*swarasa*), to prepare *swarasa*, modified methods are being adopted. In the present study, we prepared the leaf juice by different methods, including the traditional method, steaming of leaves to simulate traditional method and evaluated these juice samples for the total alkaloid content and vasicine content since the alkaloids were reported to be the active principles^13^. The main objective of the present work was to identify a method that gives juice of quality similar to that obtained by the traditional method, which can easily be adopted for commercial production.

**Fig. 1:**
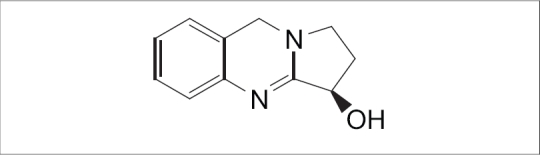
Chemical structure of vasicine.

## MATERIALS AND METHODS

For the preparation of *swarasa* from fresh leaves of *A. vasica*, leaves were collected from the plants growing in our campus. For the preparation of *swarasa* from dry leaves, the leaves were dried at 55° in a hot air oven, stored in airtight container and powdered to 40 mesh whenever required.

Tropaeolin ‘OO’ was procured from S. D. Fine Chemicals Pvt. Ltd., India. Vasicine was isolated from the leaf of *A. vasica* adopting the conventional method of acid base extraction followed by column chromatography using silica gel (60–230 #, E. Merck)^14^. It was characterized by recording UV, IR, MS and NMR spectra and melting point and comparing with the reported data^14^. The isolated vasicine was used as standard. All the chemicals used were of analytical grade.

Spotting device: Camag Linomat V Automatic Sample Spotter, (Camag, Muttenz, Switzerland); Syringe: 100 μL (Hamilton); TLC Chamber: Camag glass twin trough chamber (20 × 10 cm); Densitometer: Camag TLC Scanner 3 linked to winCATS software (Camag); TLC plates: 20 × 20 cm, precoated with silica gel 60 F_254_ TLC plate (0.2 mm uniform thickness), cut to suitable size; Spectrophotometer: UV/Vis, Shimadzu 2450 Q (Japan). Acetate buffer (pH 4.6): 5.4 g of sodium acetate and 2.66 ml of glacial acetic acid in 100 ml of double distilled water; Tropaeolin ‘OO’ solution: Saturated solution of tropaeolin ‘OO’ in double distilled water; Acid reagent: 1% v/v of concentrated sulphuric acid in methanol.

### Preparation of juice from Vasaka (*Adhatoda vasica*) leaf:

Juice from *A. vasica* leaf was prepared by different methods. Firstly traditional bolus method (modified *Put Pak Vidhi*)^12^ in which 100 g of fresh leaves of *A. vasica* were crushed using mortar and pestle, made into a bolus and it was covered with fresh leaves of *Syzigium cumini*. It was then covered with a layer (approximately 1½ inch thick) of paste of wheat flour, followed by a layer of clay paste and the ball (bolus) so obtained was dried at room temperature. The dried bolus was subjected to heat in a muffle furnace at 450°. During heating it was checked periodically and when the outer layer of the bolus became red hot and aroma of the wheat flour being baked emanated (it takes approximately 15-20 min of heating), it was taken out. The bolus was opened while hot and the leaf paste was squeezed through 4 folds of muslin cloth to obtain juice. The volume of the juice obtained was measured. This sample was coded as S-1. In the traditional method, the bolus is subjected to *laghu puta* (heat), using cow dung cakes. We modified the method slightly by heating the bolus in a muffle furnace.

The second method employed was steaming, which was carried out using two different methods. In the first method, 100 g of fresh leaves were crushed using mortar and pestle and placed in a steel vessel (without adding any water to the leaves) and heated at 121° (15 lb pressure) for 30 min. The crushed leaves were taken in 4 layers of muslin cloth and squeezed in order to obtain juice out of it. The juice obtained was measured. This sample was coded as S-2. In the second method, 100 g of fresh leaves were crushed using mortar and pestle and 100 ml of distilled water was added to it and it was subjected to heat at 121° (15 lb pressure) for 30 min. The steamed material was taken in a 4-layered muslin cloth and squeezed in order to obtain juice out of it. The juice obtained was measured. This sample was coded as S-3.

Vasaka *Swarasa* (manual) was the third method employed in which, 100 g of fresh leaves were triturated to a fine paste in a stone motor. It was taken in 4 layers of muslin cloth and squeezed by hand to take out the juice. This sample was coded as S-4. The fourth method used was Vasaka *Swarasa* (using a grinder). One hundred grams of fresh leaves were ground in a mixer/juicer with 100 ml of water and filtered through 4 layers of muslin cloth and squeezed by hand to take out the juice. This sample was coded as S-5. Finally, the juice was prepared from dry leaf powder^15^, where to 100 g of dry leaf powder, 200 ml of water was added and macerated for 24 h at room temperature. The above mixture was taken in 4 layered muslin cloth and squeezed to take out the juice. The juice obtained was measured. This sample was coded as S-6.

### Quantification of total alkaloids by spectro-photometric method^16^:

Total alkaloid content of the sample was estimated by following a reported spectrophotometric method based on the formation of coloured complex between tropaeolin ‘OO’ and alkaloids. Fifty millilitre of the juice was basified with ammonia to bring the pH to 9 and it was transferred to a separating funnel and extracted with chloroform until the aqueous extract tested negative for alkaloids when tested with modified Dragendorff's reagent^17^. The chloroform fractions were pooled, concentrated, dried over anhydrous sodium sulfate and made up to 25 ml in a volumetric flask. All the juice samples were extracted as above. These sample solutions were used for the quantification of total alkaloids and vasicine.

For the preparation of calibration curve, standard solution of vasicine (200 μg/ml) was prepared in methanol and aliquots of 0.25 ml to 1.25 ml were taken and colorimetric analysis was carried out following the method of Haussler^16^. In brief, the method is as follows: Solvent from the above aliquots was evaporated at room temperature and the residue was dissolved in 1 ml of methanol. To it 5 ml of acetate buffer and 3 ml of tropaeolin ‘OO’ solution were added and mixed well. The complex of tropaeolin ‘OO’ and alkaloids thus formed was extracted in chloroform (3 × 15 ml). The chloroform extract was dried over sodium sulfate, transferred to a 50 ml volumetric flask containing 3 ml of acid reagent and the volume was made up with chloroform. The coloured complex developed was measured at 545 nm against blank, using a double beam UV/Vis spectrophotometer. Calibration curve was prepared by plotting concentration of vasicine *vs*. absorbance.

For the estimation of total alkaloids in the samples of *Vasaka* juice (S-1 to S-6), suitably diluted aliquots were taken, the solvent was evaporated, the residue was dissolved in 1 ml of methanol and colour was developed as per method described above. Absorbance of the coloured solution was recorded at 545 nm. The amount of total alkaloids in the samples was calculated using standard curve of vasicine. The content of the total alkaloids was expressed as vasicine.

### TLC densitometric estimation of vasicine:

A stock solution of vasicine (160 μg/ml) was prepared by dissolving 4 mg of accurately weighed vasicine in methanol and making up the volume of the solution to 25 ml with methanol in a volumetric flask. The aliquots (2 to 6 ml) of stock solutions were transferred to 10 ml volumetric flasks and the volume of each was adjusted to 10 ml with methanol to obtain standard solutions containing 32, 48, 64, 80 and 96 μg/ml of vasicine.

For the preparation of calibration curve of vasicine, 10 μl each of the standard solutions of vasicine (320 to 960 ng per respective spot) were applied (band width: 6 mm, distance between the tracks: 12 mm) in triplicate on a TLC plate using the Linomat V. The plate was developed in a twin trough chamber with 10.2 ml of the mobile phase of ethyl acetate:methanol:ammonia (8:2:0.2, v/v/v) for a distance of 8 cm at 25±2° temperature and 40% relative humidity. After development, the plates were dried at room temperature in air and scanned at 298 nm in absorbance mode using deuterium lamp source of the densitometer. The peak areas were recorded. The calibration curve of vasicine was obtained by plotting peak areas *vs*. applied concentrations of vasicine.

Ten microlitres each of suitably diluted sample solutions were applied in triplicate on a TLC plate. The plate was developed and scanned as mentioned above. The peak areas were recorded and the amount of vasicine was calculated using the calibration curve.

### Validation of the method:

International Conference on Harmonization (ICH) guidelines were followed for the validation of the analytical procedure (CPMP/ICH/281/95 and CPMP/ICH/381/95). The method was validated for precision, repeatability and accuracy. Instrumental precision was checked by repeated scanning (*n* = 7) of the same spot of vasicine (480 ng/spot) and expressed as relative standard deviation (RSD). The repeatability of the method was affirmed by analyzing 480 ng/spot of vasicine, on the TLC plate (*n* = 7) and was expressed as RSD. Variability of the method was studied by analyzing aliquots of standard solution containing 320, 400, 480 ng/spot of vasicine on the same day (intra-day precision) and on different days (inter-day precision) and the results were expressed as RSD. Limit of detection (LOD) and limit of quantification (LOQ) were evaluated by applying different dilutions of the standard solution of vasicine along with the blank (methanol) and determined on the basis of signal to noise ratio. The accuracy of the method was assessed by performing recovery studies at three different levels (50, 100 and 125% addition of vasicine). Recovery and average recovery was calculated.

## RESULTS AND DISCUSSION

In several formulations of Ayurveda, juice of freshly collected plant material is used^18^, where the fresh plant material is crushed and squeezed to obtain juice called *Swarasa*. However, some plant materials like leaf of *A. vasica* and *Vitex negundo*, which do not yield juice easily by simply crushing and squeezing (believed to be due to hard cell wall), in the classical text of *Saranghdhar Samhita*^12^ an elaborate method is prescribed. We studied this traditional method and found that in this method (bolus method), steam generated from the moisture present in the leaf during heating facilitated the release of the juice containing alkaloids. In our experiments we found that the juice (*swarasa*) prepared by this method contained highest amount of alkaloids per ml of the juice. But this method is very elaborate, tedious, time consuming and difficult to handle large batches of leaf and hence not suitable for commercial application.

In the present study, we extracted the juice from *Vasaka* leaf by using six different methods. The first one (S-1) was the traditional bolus method which we compared with the method developed by us (steaming the crushed leaves without addition of water: S-2). Juice samples were also prepared by four other methods, including the one using dry leaf powder (S-6) as described by *Saranghdhar Samhita*^12^. The volume of juice obtained by processing the leaf by different methods (S-1 to S-6) is given in Table 1. We estimated the total alkaloid content by spectrophotometric method^16^ and vasicine content using TLC densitometric method (developed by us) from all the six samples (S-1 to S-6) prepared by different methods and compared.

**TABLE 1:** AMOUNT OF TOTAL ALKALOID AND VASICINE CONTENT FROM JUICE OF *ADHATODA VASICA* LEAF EXTRACTED BY DIFFERENT METHODS

Sample	Quantity of juice obtained (ml/100 gm leaf)	Total alkaloids (mg/ml)^a^	Vasicine (mg/ml)^a^
S-1	25	5.93 ± 0.55	5.64 ± 0.10
		(148.25)	(141)
S-2	25	4.05 ± 0.12	3.46 ± 0.06
		(101.25)	(86.5)
S-3	105	0.58 ± 0.01	0.33 ± 0.01
		(60.9)	(34.65)
S-4	14	1.25 ± 0.02	1.03 ± 0.01
		(17.5)	(14.42)
S-5	160	0.30 ± 0.04	0.20 ± 0.02
		(48.0)	(32.0)
S-6^b^	50	3.21 ± 0.01	1.92 ± 0.02
		(160.5)	(96)

a Mean ± SD, (*n* = 3);

b From dry leaf, In parenthesis, values calculated mg/100 g of leaf

The method adopted for the quantification of total alkaloids is the reaction between alkaloids and tropaeolin ‘OO’ to form a charge transfer complex, which can be extracted in chloroform or dichloromethane, followed by its reaction with acid reagent to give a purple coloured chromogen with λ_max_ of 545 nm^19^–^25^. The calibration curve for vasicine was found to be linear over the range of 50 to 250 μg/ml with a correlation coefficient of 0.999. This method was used for the quantification of total alkaloids from the juice obtained from S-1 to S-6 and the results of the analysis are given in Table 1.

**Fig. 2:**
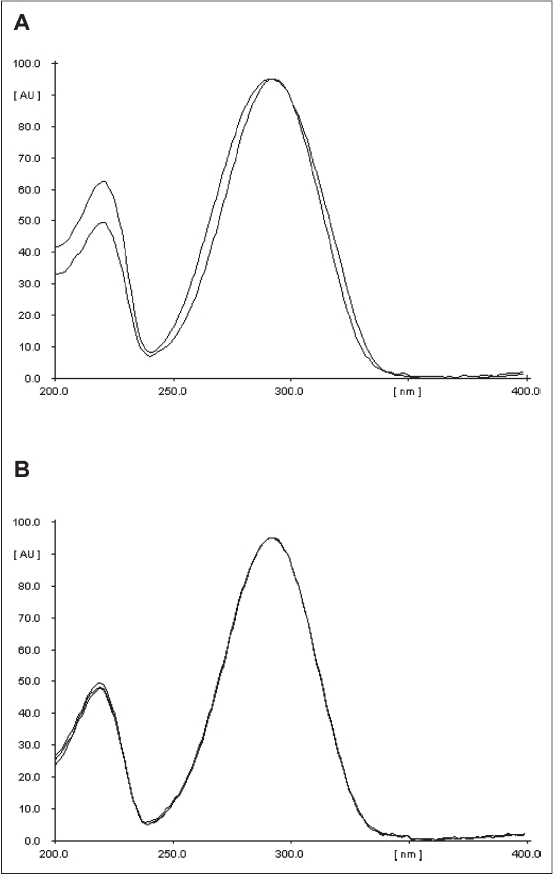
Overlay of UV absorption spectra. A. Overlay UV absorption spectra of vasicine and the corresponding band in the track of sample extract and standard; B. Overlay UV absorption spectra of vasicine in the sample track recorded at the start, middle and end positions of the band.

For TLC densitometric quantification of vasicine, preliminary TLC fingerprinting was carried out in order to optimize the mobile phase to obtain a clear-cut separation of the band of vasicine from the rest of the compounds. The optimized mobile phase resolved vasicine at R_f_ 0.45. The other compounds in the sample extracts did not interfere. The identity of the band of vasicine in the sample extract was confirmed by overlaying the ultra violet (UV) absorption spectra with that of the respective reference standard using Camag TLC Scanner 3 with winCATS software (fig. 2a). The purity of the band in the sample extract track was confirmed by comparing the absorption spectra recorded at start, middle and end positions of the band (fig. 2b).

The developed TLC densitometric method for the estimation of vasicine was validated in terms of precision, repeatability and accuracy (Table 2). The linearity range for vasicine was found to be 320-960 ng/spot, with correlation coefficient (r-value) of 0.999. The intra-day and inter-day precision expressed as RSD. (Tables 2 and 3) indicate that the proposed method was precise and reproducible. The limit of detection for vasicine was found to be 80 ng and the limit of quantification was found to be 320 ng (Table 2). The average of percentage recovery at three different levels was found to be 101.37% (Table 4).

**TABLE 2:** METHOD VALIDATION PARAMETERS FOR THE ESTIMATION OF VASICINE BY THE PROPOSED HPTLC METHOD

Parameter	Results
Accuracy (average recovery, %)	101.37
Precision (% R.S.D.)	
Repeatability (*n* = 5)	0.13
Inter-day precision (*n* = 3)	0.38
Intra-day precision (*n* = 3)	0.57
Instrumental precision (*n* = 7)	0.39
Limit of detection (ng)	80
Limit of quantification (ng)	320
Specificity	Specific
Correlation coefficient (Linearity)	0.999
Linearity range (ng/spot)	320-960
Standard deviation (%)	1.31
Linear regression equation	y = 1010.001 + 8.351×0.31
Standard error of Slope	
Standard error of Intercept	0.75

**TABLE 3:** INTRA-DAY AND INTER-DAY PRECISION OF VASICINE BY THE PROPOSED HPTLC METHOD

Amount (ng/spot)	Intra-day precision^a^	Inter-day precision^a^
320	0.57	0.42
400	0.47	0.30
480	0.68	0.25
	Mean = 0.57	Mean = 0.38

a RSD (%), *n* = 6

**TABLE 4:** RECOVERY STUDY OF VASICINE BY THE PROPOSED HPTLC-DENSITMETRIC METHOD

Amount of vasicine present in sample (μg)	Amount of vasicine added (μg)	Amount of vasicine found^a^ (μg)	Recovery a(%)	Average recovery (%)
479	238	725.43 ± 12.08	101.15 ± 1.39	101.41
479	477	947.46 ± 19.18	101.92 ± 2.07	
479	596	1104.33 ± 17.89	103.33 ± 1.64	

a Mean ± SD, *n* = 3

**Fig. 3:**
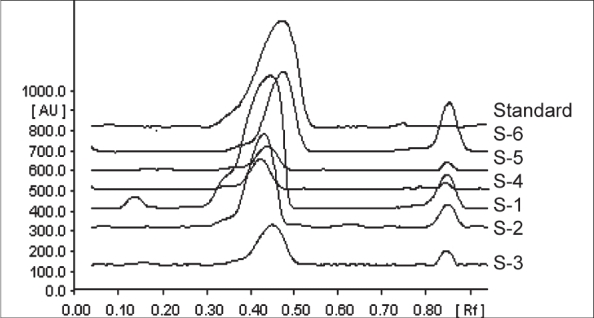
TLC densitometric chromatogram at 298 nm. TLC densitometric chromatogram at 298 nm of *Adathoda vasica* leaf juice prepared by different methods. S-1, traditional bolus method; S-2, steaming; S-3, steaming with addition of water; S-4, *swarasa* (without water) using mortar and pestle; S-5, *swarasa* (with water) using grinder; and S-6, from leaf powder.

The highest amount of alkaloids was found to be present in the leaf juice prepared by the traditional bolus method (S-1) - total alkaloids 5.93 mg/ml and vasicine 5.64 mg/ml of juice obtained. The quantity of the juice obtained by steaming without addition of water (S-2) method was same as that of the traditional method. Though the total alkaloid content (4.05 mg/ml) and vasicine content (3.46 mg/ml) of the juice obtained by this method (S-2) was a little less than that by the bolus method, it was found to be much higher than the other modified methods (fig. 3, Table 1).

Juice samples prepared by four other methods used by some manufacturers (S-3, S-4, S-5, S-6), of which S-6 was prepared from dry leaves. Their total alkaloid and vasicine content were compared (fig. 3, Table 1). The amount of total alkaloid (1.07 mg/ml) and vasicine content (0.64 mg/ml) in S-6 can not really be compared with the other methods as S-6 is obtained from dry leaf powder. However, this method (S-6) is being used by some of the manufacturers since it is convenient to handle and process dry leaf. This method though uses much more amount of leaf, yields good quality juice, as one goes by the quality of juice in terms of alkaloid content per ml (Table 1). Hence, this method can be adopted when dry leaf has to be used. The second method (S-2) can be adopted when fresh leaf is to be used. However, since the efficacy is attributed to the synergistic activity of many chemical compounds present therein, different juice samples may require to be subjected to biological evaluation also before coming to any conclusion. However, in the present case, since the alkaloids have been reported to be the active principles of *Vasaka* that are responsible for its activity, we can make a safe assumption that either the second (S-2) or the sixth (S-6) method may be adopted for commercial production depending on the availability of the fresh or dry leaf.

Comparison of the total alkaloids and vasicine content of *Vasaka* juice (*Vasa swarasa*) samples prepared by different methods reveals the importance of the traditional bolus method. Since the quantity and the quality of the juice obtained by the steaming (without addition of water) method (S-2) are comparable to that obtained by bolus method (S-1), it can be adopted by the manufacturers.

Since other alkaloids also form coloured complex with tropaeolin ‘OO’^19^–^25^, the spectrophotometric method used by us in this experiment for the quantification of total alkaloids is not specific for the alkaloids of *A. vasica* and hence, is applicable only to those formulations which have only *A. vasica* and not any other alkaloid containing drug.

The TLC densitometric method developed by us for the quantification of vasicine using HPTLC was found to be simple, accurate, precise and reproducible and can be used for quantification of vasicine from *Vasaka swarasa*, and has applicability in quantifying vasicine from herbal raw materials and formulations containing vasicine.
